# A superhydrophobic cone to facilitate the xenomonitoring of filarial parasites, malaria, and trypanosomes using mosquito excreta/feces

**DOI:** 10.12688/gatesopenres.12749.2

**Published:** 2018-04-27

**Authors:** Darren A.N. Cook, Nils Pilotte, Corrado Minetti, Steven A. Williams, Lisa J. Reimer

**Affiliations:** 1 Department of Vector Biology, Liverpool School of Tropical Medicine, Liverpool, L3 5QA, UK; 2 Department of Biological Sciences, Smith College, Northampton, MA, 01063, USA; 3 Program in Molecular and Cellular Biology, University of Massachusetts, Amherst, MA, 01003, USA

**Keywords:** Xenomonitoring, malaria, filariasis, Trypanosoma, Brugia, Plasmodium, Anopheles, superhydrophobic

## Abstract

**Background: **Molecular xenomonitoring (MX), the testing of insect vectors for the presence of human pathogens, has the potential to provide a non-invasive and cost-effective method for monitoring the prevalence of disease within a community. Current MX methods require the capture and processing of large numbers of mosquitoes, particularly in areas of low endemicity, increasing the time, cost and labour required. Screening the excreta/feces (E/F) released from mosquitoes, rather than whole carcasses, improves the throughput by removing the need to discriminate vector species since non-vectors release ingested pathogens in E/F. It also enables larger numbers of mosquitoes to be processed per pool. However, this new screening approach requires a method of efficiently collecting E/F.

**Methods: **We developed a cone with a superhydrophobic surface to allow for the efficient collection of E/F. Using mosquitoes exposed to either *Plasmodium falciparum*, *Brugia malayi* or *Trypanosoma brucei* *brucei, *we tested the performance of the superhydrophobic cone alongside two other collection methods.

**Results:** All collection methods enabled the detection of DNA from the three parasites. Using the superhydrophobic cone to deposit E/F into a small tube provided the highest number of positive samples (16 out of 18) and facilitated detection of parasite DNA in E/F from individual mosquitoes. Further tests showed that following a simple washing step, the cone can be reused multiple times, further improving its cost-effectiveness.

**Conclusions: **Incorporating the superhydrophobic cone into mosquito traps or holding containers could provide a simple and efficient method for collecting E/F. Where this is not possible, swabbing the container or using the washing method facilitates the detection of the three parasites used in this study.

## Introduction

The screening of insect vectors for the presence of DNA or RNA from human pathogens is known as molecular xenomonitoring (MX). This approach is different to transmission monitoring in that it documents exposure of a potential vector to blood-borne pathogens in the host, rather than assessing the transmission of infective stages to the host. The non-invasive monitoring of disease presence in a community is becoming increasingly important as many countries move towards the elimination of vector-borne diseases. Documenting the decline of a pathogen within a community and sensitively detecting recrudescence is essential to allow a rapid response to (re-)emergence and to prevent widespread outbreak.

The utility of MX to detect the presence of the parasite within a community has been demonstrated for lymphatic filariasis (LF) across a diverse range of locations (Ghana ^1^, Egypt ^2^, American Samoa ^3^, Papua New Guinea ^4^ and Sri Lanka ^5^), with different species of vectors ( *Anopheles*, *Aedes* and *Culex* species) and parasites ( *Wuchereria bancrofti*, *Brugia malayi*, and *Brugia timori* ^6, 7^) *.* The Global Programme to Eliminate LF aims to eliminate the disease as a public health problem by 2020 using the strategy of at least five rounds of annual mass drug administration (MDA) to interrupt transmission of the parasite. Following MDA scale down, transmission assessment surveys (TAS) will be conducted to screen for the presence of adult worm antigens ( *W. bancrofti*) or host antibodies ( *B. malayi)* in school children to determine whether exposure has occurred post-MDA ^8^. Conducting TAS can be logistically challenging, expensive ^9^ and is dependent on strong community engagement and acceptance if it requires repeated sampling ^10^. In some settings, after many rounds of MDA, TAS may lack the sensitivity required to accurately detect ongoing transmission ^11^. As an example, having passed two TAS, MX identified areas of localised transmission in American Samoa ^12^. Using MX to complement TAS may enable targeted interventions that reduce the likelihood of recrudescence. An unsuccessful TAS indicates transmission rates may exceed the critical threshold, leading to the development of new, patent, infections requiring the community to undertake additional rounds of MDA. Since MX has the potential to provide real-time, non-invasive and cost-effective surveillance of the human population, a positive sample could trigger a rapid, targeted response, preventing further infections and reducing the need for additional rounds of MDA.

As control programmes for other vector-borne diseases, such as Human African Trypanosomiasis ^13^ and malaria ^14^, continue to make progress towards elimination, MX could have a similar role to play in the monitoring of community infection levels by using insect vectors as ‘flying syringes’ ^15^. Sensitive detection of the causative agents of malaria and trypanosomiasis has been demonstrated using MX ^16, 17^. MX may help malaria control programmes to direct vector control measures to specific areas of potential transmission or to implement surveillance in outbreak prone areas, such as border crossings. It could also aid the mapping of multidrug resistant (MDR) *Plasmodium* haplotypes, allowing the identification and monitoring of geographical hotspots of MDR malaria, leading to targeted measures aimed at containing its distribution ^18^. The utility of MX is not restricted to parasitic disease, since viruses have also been detected in insect vectors ^19, 20^, leading to the possibility that MX could provide an early alert for the increase of the virus within a community too ^21^.

Despite these promising applications, MX has yet to be adopted as standard practice for several reasons. For LF for example, in areas of low transmission/low parasite density, MX requires the collection and processing of large numbers of mosquitoes ^1, 22, 23^. Although pooling mosquitoes can aid the screening effort, the number of mosquitoes that can be combined is limited to 25, since larger pools decrease the sensitivity of parasite detection ^24^. The need to screen large numbers of mosquitoes in small pool sizes reduces the cost-effectiveness of MX.

The development of a screening method that uses excreta/feces (E/F) collected from mosquitoes, rather than whole carcasses is an elegant solution to this problem. The screening of E/F allows the detection of parasite DNA from the voided material of up to 500 mosquitoes ^16^ at a time. Collecting E/F also allows the recovery of parasite DNA from non-competent vector species, since the parasites are unable to establish within the vector and are released in the E/F post-bloodmeal ^25^. The use of much larger pools of mosquitoes and the lack of sorting required to isolate competent vectors should greatly reduce the cost, time and effort; therefore overcoming some of the current barriers to the large-scale implementation of MX for disease monitoring. Furthermore, mosquitoes collected for other surveillance purposes could be screened for pathogens without compromising the sample. This would provide the opportunity for joint surveillance of pathogens of interest in the community.

Although detection of parasite DNA in E/F has shown promise in laboratory settings ^16^, an efficient method of collecting E/F samples from field-caught mosquitoes still needs to be developed. One consideration is the method of mosquito collection employed, as this may dictate the most appropriate method to collect E/F, due to the physical constraints that either a trap or mosquito housing container may impose. The aim of this paper is to further validate the E/F collection approach by feeding mosquitoes a standard concentration of each parasite to assess different methods of collection that may be implemented in the field.

## Methods

### Mosquito rearing and blood-feeding

*Anopheles gambiae* were reared from eggs to adults and housed under standard conditions (26°C and 80% relative humidity). Three to seven day old mosquitoes were sugar starved for 18 hours before each assay to ensure successful blood feeding. After starvation, mosquitoes were fed either a standard bloodmeal (human blood, obtained from the blood bank) or a bloodmeal spiked with a parasite of known density using the Hemotek feeding system ( *B. malayi* and *Trypanosoma brucei brucei*) or using a glass feeder ( *Plasmodium falciparum*). Twenty-four hours after feeding, mosquitoes were added to paper cups (10 per cup) covered with netting, with sugar-soaked cotton wool placed on top. Three cups of exposed mosquitoes and one cup of control mosquitoes per E/F collection method were held for 24 hours, before transferring to fresh cups for a further 24 hours. For the testing of E/F from individually-housed mosquitoes, the same protocol was used, except that after *P. falciparum* exposure, one mosquito was added per paper cup instead of 10.

### Parasites

A single, biologically relevant density was chosen for each parasite to ensure that each mosquito would ingest parasite material to enable comparison between collection methods, without the limits of detection affecting the results. For *B. malayi* exposures, microfilariae were obtained from in-house infected gerbils ( *Meriones unguiculatus*) by intraperitoneal lavage ^26^ and added to human blood to a final concentration of 5000 mf/ml (100 mf/20 µl). *T. b. brucei* bloodstream form MCRO/ZM/72/J10 CLONE 1 ^27^ originally isolated from an infected rat, were added to human blood to give a final concentration of 2.2×10 ^6^ parasites/ml. For *P. falciparum*, 3 ml of red blood cells with a trophozoite parasitaemia of 0.4% (3D7 strain) was combined with 3 ml of human serum, to give an estimated parasite load of 20,000/µl. To test the sensitivity of parasite DNA detection from E/F, mosquitoes were fed blood with either a 0.4% or 0.1% *P. falciparum* parasitaemia, before being individually housed in cups containing a superhydrophobic cone.

### Excreta/feces collection methods

Four different methods were used to collect the mosquito E/F. Two methods utilise superhydrophobic cones placed inside paper cups, whereas the other two methods did not require the superhydrophobic cone.

**Figure 1.**
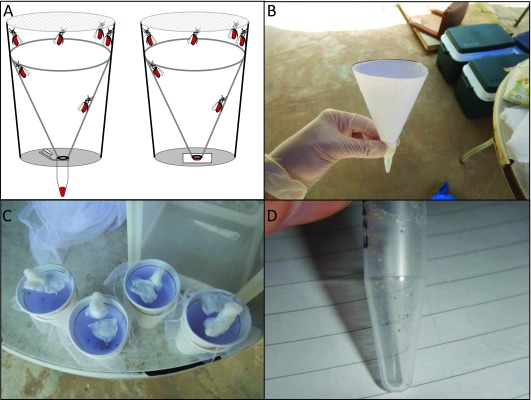
Schematic of the superhydrophobic cone and images from the field collections. ( **A**) Schematic of the collection cups with the superhydrophobic cone used to collect excreta/feces (E/F) into a tube (left) or onto FTA card (right). From the field site - ( **B**) the superhydrophobic cone being assembled, ( **C**) wild-caught mosquitoes in the collection cups with superhydrophobic cones, ( **D**) the resultant E/F in a collection tube.

***Superhydrophobic cone collection methods.*** The first two methods both required the construction of paper cones with a superhydrophobic surface. To create the cone, A4 printer paper, cut to form a semi-circle was coated with NeverWet® (Rust-Oleum, Durham, UK) as per the manufacturer’s instructions. Superhydrophobic cones were washed with water in a non-abrasive manner, to remove any excess coating, then placed inside paper cups to allow the E/F to be deposited on either a small square of Whatman (GE Health Sciences, Buckinghamshire, UK) FTA card or into a 1.5 ml tube at the bottom of the cup ( Figure 1A).

Following the transfer of mosquitoes at each 24-hour timepoint, the FTA cards or 1.5 ml tubes were removed from the bottom of the used cups. FTA cards containing E/F were stored individually in sealed plastic bags containing silicon beads, to protect samples from moisture. The 1.5 ml tubes were sealed and stored at -20°C.

### Alternative collection methods

In the absence of a superhydrophobic cone inside the paper cup, mosquito E/F is deposited directly on to the interior surface of the cup. This E/F can be collected by either direct washing of E/F, or by swabbing the interior of the cup followed by DNA extraction from the swab.

***Swab collection method.*** After the removal of mosquitoes, the paper cup was carefully opened along the seal to enable the swabbing of all internal surfaces. A small volume of nuclease-free water (~10 µl) was added to the surface of the swab (IsoHelix DNA buccal swab). The wetted surface of the swab was used to resuspend and collect any E/F material in the cup, before using the dry side of the swab to collect any remaining material. Swab heads were removed and placed into 2ml tubes before storing at -20°C.

***Wash collection method.*** Deposits of E/F material were resuspended in 100 µl nuclease-free water using a 200 µl pipette and gentle drawing up and down of the suspension. The 100 µl of resuspended material was added to 100 µl of ATL buffer in a 1.5 ml tube and stored at -20°C.

### Parasite detection

For all four collection methods, DNA was extracted using the QIAamp® DNA Micro Kit (QIAGEN, Manchester, UK). E/F samples on FTA card were processed following the ‘Isolation of Genomic DNA from Dried Blood Spots’ protocol from the manufacturer’s handbook. For the E/F samples collected in the 1.5 ml tubes, the ‘Isolation of Genomic DNA from Small Volumes of Blood’ protocol from the manufacturer’s handbook was followed with the following modifications to the reagent volumes: 180 µl of buffer ATL, 20 µl Proteinase K, 200 µl Buffer AL, 100 µl Ethanol. For swabs, DNA was extracted using the ‘Isolation of Total DNA from Surface and Buccal Swabs’ protocol except that the 56°C incubation step was increased to 3 hours. For wash samples, the ‘Isolation of Genomic DNA from Small Volumes of Blood’ protocol was followed, beginning at the addition of proteinase K, with the following amendments to reagent volumes: 20 µl Proteinase K, 200 µl Buffer AL, 100 µl Ethanol. All DNA extraction samples were eluted in 20 µl of nuclease-free water.

Amplification and detection of *B. malayi* DNA using real-time PCR was performed as described previously ^28^, with modifications to the reaction mixture: 1x TaqPath ProAmp Master mix (Applied Biosystems, Foster City, CA, USA), 0.4 µM of each primer, 0.125 µM of probe and 2 µl of DNA to give a final volume of 10 µl.

*P. falciparum* was detected as described by Kamau *et al.* ^29^ using only the *P. falciparum* specific primers and probe. Each reaction contained: 1x TaqPath ProAmp Master mix, 0.4 µM of each primer, 0.2 µM of probe and 2 µl of DNA to give a final volume of 10 µl.

The probe-based PCR cycling conditions were the same for both *B. malayi* and *P. falciparum* detection and were as follows: 95°C for 10 minutes followed by 40 cycles of 95°C for 15 s; 60°C for 60 s.

*T. b. brucei* DNA was detected using conventional PCR as detailed by Cunningham *et al.* ^17^, using the TBR primers designed by Kazibwe ^30^.

### Cone reusability testing

To assess the reuse of the superhydrophobic cone, mosquitoes exposed to a bloodmeal containing *P. falciparum* 2 days prior were added to cups containing superhydrophobic cones as described above (10 mosquitoes per cup, total of 3 cups). After 24 hours, mosquitoes were removed and 1.5 ml tubes containing the collected E/F were stored at -20°C until DNA extraction. The inner surfaces of the cones were washed using a Pasteur pipette to provide a non-abrasive stream of distilled water from around the rim of the cone. After washing, 200 µl of nuclease-free water was added drop-wise around the top of the inside of the cones and collected in 1.5 ml tubes at the bottom, to mimic the collection of mosquito excreta. The washing process was repeated and a further sample collected for each cone before storing at -20°C. Identical collections were made using mosquitoes fed on unexposed blood to act as a control. DNA extraction and real-time PCR was performed to test for the presence of *P. falciparum*.

The above process was repeated, but instead of collecting E/F from exposed mosquitoes, 10 µl of DNA extracted from previous E/F collections was diluted 1 in 10 and 100 µl of this was added drop-wise around the top of each cone (three cones in total) and collected in a 1.5 ml tube at the bottom. The washing and sample collections were carried out as above and all samples were stored at -20°C, until real-time PCR was performed to test for the presence of *P. falciparum*.

To test cones for the retention of superhydrophobicity, three cones were washed ten times, as detailed above, before water was added drop-wise around the top of the inside of the cones using a Pasteur pipette. If no droplets adhered to the cone or caused wetting to the surface, then the cone was considered to have retained superhydrophobicity.

### Data analysis

Real-time PCR data were captured using Bio-Rad CFX manager version 1.5 software, before exporting to Microsoft Excel 2016 to calculate mean Ct values.

## Results

Mosquitoes were exposed to one pathogen per assay. Deposited material was collected on days 2 and 3 post-bloodmeal using each of the four E/F collection methods. All four methods employed to collect E/F allowed the detection of DNA from all three parasites ( Table 1). Using the superhydrophobic cone to collect E/F material in a 1.5 ml tube offered the highest rate of detection across the three different parasites ( Table 1), with 16 out of 18 samples showing positive for the presence of parasite DNA, followed by the wash method (14/18), superhydrophobic cone + FTA (13/18) and the swab method (12/18).

**Table 1.** Detection of parasite DNA for each of the four different excreta/feces collection methods.

	Detection of DNA (positive detection/number of samples)		
Collection method	*Brugia* *malayi*	*Trypanosoma* *brucei*	*Plasmodium* *falciparum*
Superhydrophobic cone + tube	5/6	6/6	5/6
Superhydrophobic cone + FTA	3/6	4/6	6/6
Wash method	4/6	4/6	6/6
Swab method	2/6	4/6	6/6

The detection of *B. malayi* DNA was highly variable between the different collection methods, ranging from 2 out of 6 samples showing positive for the swab method compared to 5 out of 6 for superhydrophobic cone + tube. The detection of both single-cell parasites proved to be more consistent between methods, highlighting the possible need to tailor the collection method to the type of parasite that is being screened for, as well as the type of mosquito collection method that is used. We assessed the samples from the *P. falciparum* exposure to determine the total DNA collected. The mean values for each day were highest for the for superhydrophobic cone + tube (23 – 385 ng/μl) and cone + FTA (7 – 216 ng/μl), compared to the wash method (10 –16 ng/μl) and swab method (6 – 8 ng/μl). Total DNA did not reflect the likelihood of parasite detection, possibly due to the greater presence of DNA from the mosquito and ingested blood obscuring the smaller quantity of parasite DNA.

Some observations on the practicalities of each of the methods employed are presented in Table 2.

**Table 2.** Advantages and disadvantages of each excreta/feces (E/F) collection method.

Collection method	Advantages	Disadvantages
Superhydrophobic cone + tube	- Collects all E/F into 1.5 ml tube ready for DNA extraction	- E/F can sometimes be prevented from entering tube by loose cotton fibres or dead mosquitoes - Requires mosquitoes to be housed in a container/trap suitable for the cone
Superhydrophobic cone + FTA	- May protect DNA from degrading and allow for longer term storage. Adaptable to detection of virus RNA	- Loose cotton fibres or dead mosquitoes stop E/F from being absorbed into the FTA card - Areas containing E/F must be punched out for processing - Requires mosquitoes to be housed in a container/trap suitable for the cone
Wash	- Majority of E/F collected and added to storage buffer which may allow for longer term storage	- Time consuming/labour intensive - requires the surface of the container housing the mosquitoes to be waterproof - Difficult to see excreta, which is relatively colourless
Swab	- Requires no additional equipment for collection other than the swab	- Not all E/F material is recovered from the swab during DNA extraction - One swab has limited capacity to collect E/F. Containers with greater amount of deposits may require several swabs

To investigate the possibility of reusing the superhydrophobic cones, cones (n=3) were tested for contamination after housing *P. falciparum* exposed mosquitoes. After non-abrasive washing with distilled water, 100 µl of nuclease-free water was added drop-wise around the top of the cone and collected. This washing process was repeated and after DNA extraction from the collected E/F and both collected washes, samples were tested using real-time PCR ( Table 3). Ct values for the collected E/F material showed the presence of *Plasmodium* DNA as expected ( Table 3 - Collection 1); however no samples collected after either of the wash steps were positive for parasite DNA. The experiment was repeated but instead of housing mosquitoes in cups containing cones, DNA extracted from the E/F of exposed mosquitoes was added directly to the cones. The initial collection of the DNA run-through was positive for parasite DNA, and all subsequent samples collected post-washing were negative. These results indicate that after a simple washing step, the superhydrophobic cones are free from contaminating DNA and can be reused.

**Table 3.** Reusability of the superhydrophobic cone.

	Ct values		
Sample	Collection 1	Collection 2 (after first wash)	Collection 3 (after second wash)
E/F cone 1	29.7	*-ve*	*-ve*
E/F cone 2	29.5	*-ve*	*-ve*
E/F cone 3	29.2	*-ve*	*-ve*
DNA Cone 1	31.3	*-ve*	*-ve*
DNA Cone 2	30.9	*-ve*	*-ve*
DNA Cone 3	31.4	*-ve*	*-ve*
Negative Control	*-ve*	*-ve*	*-ve*

After successfully demonstrating the reuse of the superhydrophobic cone, we tested the performance of the cone when used to collect E/F from individually-housed mosquitoes, exposed to a lower density of infection. We detected *P. falciparum* DNA in the E/F from each of the 12 mosquitoes that were housed individually ( Table 4), even though six mosquitoes were fed on blood containing a fourfold reduction in parasitaemia (0.1%). Detection mainly occurred in the E/F samples collected from 48–72 hours post-bloodmeal ( Table 4 – Day 3), which coincided with the peak amount of E/F collected in the 1.5 ml tubes. As expected, the higher parasitaemia led to lower Ct values, indicating a greater quantity of parasite DNA present in these samples.

**Table 4.** Detection of *Plasmodium falciparum* DNA from the excreta/feces of individual mosquitoes collected using superhydrophobic cones.

	Ct value	
Sample	Day 2	Day 3
**0.10%**		
A	*-ve*	34.5
B	*-ve*	35.7
C	*-ve*	36.9
D	34.9	35.3
E	35.8	*-ve*
F	*-ve*	36
**0.40%**		
G	*-ve*	32.6
H	*-ve*	30.2
I	*-ve*	31.6
J	*-ve*	32.4
K	*-ve*	34
L	*-ve*	33.6
Controls	*-ve*	*-ve*

## Discussion

Molecular xenomonitoring (MX) is a sensitive tool to detect the presence of infection within a community, particularly for diseases that cause a high number of asymptomatic infections, where there is low treatment seeking behaviour, and late onset of symptoms. However, several challenges exist that prevent the widespread use of MX for post-MDA surveillance. One such barrier is the limit on the number of mosquitoes that can be pooled for analysis, increasing the quantity of reagents, time and labour required to process samples and thus increasing the cost. Using E/F released from mosquitoes, instead of whole mosquitoes, greatly improves the ratio of parasite to mosquito DNA and allows the screening of at least twenty times the number of mosquitoes than the standard approach ^16^. We cannot say whether using E/F also has the advantage of lesser PCR inhibition compared to whole mosquitoes, since we used a DNA extraction method likely to remove inhibiting substances that may be present in blood and therefore also in E/F. The incorporation of a simple, low-cost, homemade adaptation to mosquito collection cups, in the form of a superhydrophobic cone, enabled the consistent detection of *B. malayi*, *P. falciparum* and *T. b. brucei*, with minimal processing of the E/F samples. Although the homemade cone is adaptable to a range of different containers, in situations where the use of the cone is not practical, we have shown that both swabbing or employing the wash method to collect E/F facilitates the detection of parasite DNA. Furthermore, the method employed here was sensitive enough to consistently detect the presence of *P. falciparum* DNA in E/F from individual mosquitoes. In our pilot studies, we detected *T. b. brucei* DNA in E/F from individual mosquitoes (unpublished data) and similar results have been reported for *B. malayi* detection ^25^.

Coating a sheet of paper with an inexpensive superhydrophobic layer before crafting into a cone shape allowed the entire amount of deposited E/F to be collected for DNA extraction, without requiring further processing, unlike the wash method or using a swab. This novel coating ensures that all E/F rolls quickly to the bottom of the cone to be collected in a 1.5 ml tube or to be absorbed onto an FTA card. Alongside our team’s field collections, the superhydrophobic cone method was tested in typical field conditions, using wild-caught mosquitoes to collect the E/F over a 24-hour period, without causing mortality to the mosquitoes. Although the cones were not tested using parasite-exposed mosquitoes, we found that the performance of the cones in the field matched that of our laboratory trials in terms of depositing the E/F into tubes ( Figure 1B–D). Our laboratory based tests show that the introduction of a simple washing technique ensures that the cone is free from contaminating parasite DNA. The lack of contamination is likely due to water-based droplets immediately running off the surface of the cone. Any tiny droplets that remain are removed during washing, since superhydrophobic surfaces are often referred to as self-cleaning ^31^, with the addition of water enabling the removal of dust and small particles from the surface. In our experience, at least ten washing cycles of the cone can be conducted without disruption to the superhydrophobic properties, allowing for repeated reuse, further improving the cost-effectiveness of E/F collection. The number of times a cone can be reused will depend upon the handling between use, as harsh surface abrasion or exposure to certain detergents may diminish the superhydrophobicity of the cone. The washing steps allow the user to confirm that the superhydrophobic properties of the cone are intact prior to reuse.

We envision that the use of a superhydrophobic cone or other shaped surface could be integrated into a passive trap to allow the easy recovery of E/F from large numbers of mosquitoes with minimal cost or labour. In situations where such a device may not be considered practical, employing the wash or swabbing method to collect E/F is likely to be similarly effective (particularly for single-cell parasites) albeit more time consuming. The reduction in the detection of *B. malayi* DNA when E/F was collected by swabbing may be explained by the fact that swabbing left a greater amount of E/F residue still on the swabbed surface and not all the absorbed material was removed from the swab during DNA extraction. With less E/F material being available for DNA extraction, and therefore fewer copies of parasite DNA present in the PCR reaction, the chance of false negatives increases. That this only occurred for *B. malayi* could be due to a lower amount of parasite DNA present in these samples to begin with, when compared to *P. falciparum* and *T. b. brucei*.

Our future work will focus on determining the sensitivity of detection for *B. malayi*, *P. falciparum* and *T. b. brucei* when using the superhydrophobic cone to collect E/F. Also, we will examine whether vector-borne viruses can be detected in the E/F of both vectors and non-vectors and to discover if MX can be extended to pathogens that are not transmitted by vectors at all, but that may be picked up from a human host during bloodfeeding. Here we show that DNA from *T. brucei* is detected in the E/F of *A. gambiae*, which is not a vector, but would ingest and excrete the parasites while blood feeding on an infected individual. If non-vector-borne diseases could be detected in E/F, this would allow the screening of a much broader range of pathogens within a community using the MX approach. If this is the case, it should be possible to develop a PCR-based approach that could simultaneously screen for a battery of pathogens, giving valuable, real-time information on the presence and transmission of disease within a specific location. This information could be used to initiate more comprehensive mapping of disease prevalence, instigate test-and-treat for applicable pathogens or to implement vector control interventions to specific areas to stop transmission.

## Data availability

Raw data are available on OSF: http://doi.org/10.17605/OSF.IO/9KQJR ^32^.

Data are available under the terms of the Creative Commons Zero “No rights reserved” data waiver (CC0 1.0 Public domain dedication)
