# Supplementary material for: A superhydrophobic cone to facilitate the xenomonitoring of filarial parasites, malaria, and trypanosomes using mosquito excreta/feces
Source: Gates Open Res. 2018 Apr 27;1:7. Originally published 2017 Nov 6. [Version 2] doi: 10.12688/gatesopenres.12749.2 (PMC5781187; doi:10.12688/gatesopenres.12749.2)
Supplement: Peer review history

# Peer review: Referee response for version 1

**DOI:** 10.21956/gatesopenres.13809.r26156

**Published:** 2018-01-19

**Authors:** Warwick N. Grant

**Affiliations:** 1 Department of Animal, Plant and Soil Sciences, La Trobe University, Bundoora, Vic, Australia

- This study makes important distinction in Introduction that the aim is to measure disease prevalence and not transmission potential i.e. have vectors fed on an infected person rather than have ingested microfilaria matured to infective larvae.
- The basic premise of this work is that the excreta of engorged female mosquitos will contain sufficient parasite DNA for detection, and has been demonstrated previously.
- This manuscript addresses the need to “operationalize” earlier research by developing a simpler, more routine method for excreta collection.
- In the Methods section, under the subheading “Excreta/faeces collection methods” there are 4 methods listed. I found the organisation of this section slightly confusing because the first section (headed “Superhydrophobic cone collection methods” starts with a sentence stating that there are 4 methods, only two of which utilise superhydrophobic cones. It is not entirely clear whether the last two methods described (swab & wash collection) are washing and swabbing of a hydrophobic cone, or of the inside surface of a paper cup without the addition of a cone. An alternative arrangement that makes this more obvious might be…

Excreta/faeces collection methods

 Four different methods were used to collect mosquito E/F, two of which utilise superhydrophobic cones inside paper cups and two of which do not require superhydrophobic cones.

***Superhydrophobic cone collection methods.*** To create the cone…. (then follow with the description of the cone methods as per the original text).

***Alternative collection methods***. In the absence of a superhydrophobic cone insert into the paper cup, mosquito E/F is deposited directly on to the interior surface of the cup. This E/F can be collected by either direct washing of E/F, or by swabbing the interior of the cup followed by DNA extraction from the swab. … (then follow with the description of these two methods as per the original text). 

  

- The conclusions reached by the authors are supported by the data they present. As the authors point out, this is a laboratory based study utilising mosquitos fed pathogen infected blood under controlled conditions, and the utility of the method for field based application remains to be determined.
- With respect to citation of relevant literature, there is a more recent review of nucleic acid based xenomonitoring that could be cited (Minetti et al. (2016) ^1^) and there is literature on virus xenomonitoring in mosquitos via collection of saliva deposited during sugar feeding (Hall-Mendelin et al. (2010) ^2^). The latter paper has been cited more than 25 times for xenomonitoring of virus pathogens and, although not tested for filarial parasites, may be worth including given that the authors suggest that E/F analysis may be applied to viruses in addition to filarial and protozoan parasites.

I have read this submission. I believe that I have an appropriate level of expertise to confirm that it is of an acceptable scientific standard.

## References

[1] MINETTICLACOURSEEREIMERLSTOTHARDJ: Focusing nucleic acid-based molecular diagnostics and xenomonitoring approaches for human helminthiases amenable to preventive chemotherapy. *Parasitology Open*.2016;2: 10.1017/pao.2016.13 10.1017/pao.2016.13

[2] Hall-MendelinSRitchieSAJohansenCAZborowskiPCortisGDandridgeSHallRAvan den HurkAF: Exploiting mosquito sugar feeding to detect mosquito-borne pathogens. *Proc Natl Acad Sci U S A*.2010;107(25) : 10.1073/pnas.1002040107 11255-9 10.1073/pnas.1002040107 20534559PMC2895145

# Peer review: Referee response for version 1

**DOI:** 10.21956/gatesopenres.13809.r26053

**Published:** 2017-11-16

**Authors:** Dziedzom K. de Souza

**Affiliations:** 1 Department of Parasitology, Noguchi Memorial Institute for Medical Research, University of Ghana, Accra, Ghana

Xenomonitoring presents opportunities for assessing the presence/absence of infections in populations through the testing of hematophagous insects for specific pathogens of interest. The study presented here by Cook and colleagues, highlights important developments in the field of xenomonitoring, testing new tools to enhance the applicability of MX as a public health tool. The paper is well written and acceptable for indexing. However, there are some comments/clarification which could improve the clarity and quality of the manuscript.

 Comments:

- The mosquitoes in the laboratory were fed with blood meals containing very high parasite load, which may not always be found in the field. It would also have been appropriate to test the efficiency of the cones using low parasite load bloodmeals. This will also enable the determination of any correlation between the parasite counts in the blood meal and the Ct values obtained. However, I do appreciate that it is the superhydrophobic cone that is being evaluated and not really the use of mosquito excreta.
- Section under “Cone reusability testing”. I think the washing procedure here is very important for replicability and should be further expanded. Too much washing may lead to destruction of the cone, while too little may leave DNA that could contaminate future samples. The term “gentle washing” is relative.
- In the results section, even though samples were tested after washing, and no samples found positive for parasite DNA, what is the possibility that DNA patches were not missed in the process? I guess my difficulty is with the washing procedure. When the authors state that “100 μl of nuclease-free water was added dropwise around the top of the cone and collected” what do they mean? Was all the surface covered? I am just trying to picture the process. Assuming this drawing represents the cone, and the lines with arrows represent the 100 μl of nuclease-free water, would it be right to say that sections (between the arrows) may have been missed?
- In the discussion, Section starting “Using E/F released from mosquitoes, instead of whole mosquitoes, greatly improves the ratio of parasite to mosquito DNA and allows the screening of at least twenty times the number of mosquitoes than the standard approach” it might be worth mentioning other advantages/ disadvantages to using mosquito excreta compared to whole mosquito extractions. One typical example is the role of insect cuticle in inhibiting PCR reactions.
- Would the chemical used in coating the cone have any effect on DNA stability? In this study, high parasite load bloodmeal were used, which may imply high DNA recovery. But should the opposite be the case, would enough DNA be recovered for testing?
- Last but one paragraph of discussion “we envisage that the use of a … into a passive trap to allow…”. Please explain how this will be used in field conditions. Practical examples may be useful. Can large numbers of mosquitoes be collected using aspirators or other methods without large cost or labor inputs?
- “The reduction in the detection of B. malayi DNA when E/F was collected by swabbing may be explained by the fact that swabbing left a greater amount of E/F residue still on the swabbed surface”. This could be evaluated by testing the surface (cutting it out) for DNA.
- Tables 1 and 4. If possible, provide details on the concentration of DNA recovered by each method, and for each parasite species. This might be useful in estimating the parasite detection limits.

I have read this submission. I believe that I have an appropriate level of expertise to confirm that it is of an acceptable scientific standard.

# Response

**Published:** 2017-11-27

**Authors:** Lisa Reimer

Our response to reviewer 1 comments appear below in italics

- The mosquitoes in the laboratory were fed with blood meals containing very high parasite load, which may not always be found in the field. It would also have been appropriate to test the efficiency of the cones using low parasite load bloodmeals. This will also enable the determination of any correlation between the parasite counts in the blood meal and the Ct values obtained. However, I do appreciate that it is the superhydrophobic cone that is being evaluated and not really the use of mosquito excreta.

*We agree that it will be important to test the lower limits of detection before scaling up the approach. For this study, we wanted to compare different collections methods while keeping the potential for variability low between exposed mosquitoes. We have clarified the objective and also included this as a description of our future work.*

- Section under “Cone reusability testing”. I think the washing procedure here is very important for replicability and should be further expanded. Too much washing may lead to destruction of the cone, while too little may leave DNA that could contaminate future samples. The term “gentle washing” is relative.

*We have clarified this in the text to mean “non-abrasive washing”. The coating is durable when washed according to the manufacturer’s instructions - if water is applied without abrasive force the coating will remain undamaged. *

- In the results section, even though samples were tested after washing, and no samples found positive for parasite DNA, what is the possibility that DNA patches were not missed in the process? I guess my difficulty is with the washing procedure. When the authors state that “100 μl of nuclease-free water was added dropwise around the top of the cone and collected” what do they mean? Was all the surface covered? I am just trying to picture the process. Assuming this drawing represents the cone, and the lines with arrows represent the 100 μl of nuclease-free water, would it be right to say that sections (between the arrows) may have been missed?

*It is difficult to be certain as to whether the whole surface was contacted by the water, as when the droplets hit the top of the cone, they rapidly roll or bounce to the bottom of the cone and into the tube. We tried to increase the contact with the surface area of the cone by depositing the droplets at an angle – leading to a corkscrew pattern of rinsing the cone. Since the cone tapers to a point we are confident that the DNA (or the excreta from mosquitoes) and the subsequent rinsing both passed over the same areas near the bottom of the cone.*

- In the discussion, Section starting “Using E/F released from mosquitoes, instead of whole mosquitoes, greatly improves the ratio of parasite to mosquito DNA and allows the screening of at least twenty times the number of mosquitoes than the standard approach” it might be worth mentioning other advantages/ disadvantages to using mosquito excreta compared to whole mosquito extractions. One typical example is the role of insect cuticle in inhibiting PCR reactions.

*We have added a statement to say the DNA extraction approach used within the paper should remove proteins that inhibit PCR (referencing the Pilotte et al. 2016 paper). However we can’t confirm the absence of inhibitory products since these may be present in the blood (and possibly the faeces as well).*

- Would the chemical used in coating the cone have any effect on DNA stability? In this study, high parasite load bloodmeal were used, which may imply high DNA recovery. But should the opposite be the case, would enough DNA be recovered for testing?

*Before the first use, cones were gently washed to remove any excess coating that might contaminate the excreta sample. We failed to describe this adequately in the manuscript, but have added further details. The general improvement in the detection of parasite material compared to swabbing or washing indicates that when using the same parasite load, the cone is likely to produce improved detection, so DNA stability is unlikely to be affected for the short periods of collection employed here. We cannot say whether DNA stability would be affected over a longer period of time, but contact time with the coating is very short, reducing the likelihood of any interference*.   

- Last but one paragraph of discussion “we envisage that the use of a … into a passive trap to allow…”. Please explain how this will be used in field conditions. Practical examples may be useful. Can large numbers of mosquitoes be collected using aspirators or other methods without large cost or labor inputs?

*This is exactly what we’d like to explore with future research (or inspire others to develop passive trap ideas), though we cannot yet comment on what methods would be most appropriate to keep costs down* 

- “The reduction in the detection of B. malayi DNA when E/F was collected by swabbing may be explained by the fact that swabbing left a greater amount of E/F residue still on the swabbed surface”. This could be evaluated by testing the surface (cutting it out) for DNA.

*This is a qualitative observation rather than a quantitative result – the swab quickly became saturated and multiple swabs would be needed to pick up all the material.*

  

- Tables 1 and 4. If possible, provide details on the concentration of DNA recovered by each method, and for each parasite species. This might be useful in estimating the parasite detection limits.

*Good idea, we have quantified the amount of DNA in each sample and will present it in the results. Although we can’t be sure whether we have parasite, host or mosquito DNA in the sample, it does illustrate the ability of the methods to recover deposited material.*
